# Oral symptoms potentially associated with mild-to-moderate COVID-19 in tobacco users

**DOI:** 10.18332/tid/186531

**Published:** 2024-05-13

**Authors:** Hanaa E. Alkharobi, Manar M. Alzahrani, Shatha Bamashmous, Abdullah Alghamdi

**Affiliations:** 1 Department of Oral Biology, College of Dentistry, King Abdul-Aziz University, Jeddah, Saudi Arabia; 2 Department of Oral and Maxillofacial Prosthodontics, Faculty of Dentistry, King Abdulaziz University, Jeddah, Saudi Arabia; 3 Department of Periodontology, Faculty of Dentistry, King Abdulaziz University, Jeddah, Saudi Arabia; 4 Ministry of Health, Jeddah, Saudi Arabia

**Keywords:** COVID-19, oral manifestations, tobacco products, ageusia

## Abstract

**INTRODUCTION:**

Coronavirus disease (COVID-19) is a worldwide infection characterized by various symptoms. Few studies have examined its oral manifestations. However, there is insufficient information on the oral manifestations of patients with COVID-19 who use tobacco products. Therefore, this cross-sectional study investigated oral symptoms of tobacco-using patients with mild-to-moderate COVID-19.

**METHODS:**

This study used a convenience sample of non-hospitalized patients (aged ≥18 years) with mild-to-moderate COVID-19 diagnosed by polymerized chain reaction (PCR). This study excluded pregnant or lactating women or patients with serious COVID-19 complications, including those who required hospitalization or were on specific medications (antiviral, corticosteroid, antimicrobial, or immunosuppressive). Oral examinations were performed, including labial, buccal, and gingival mucosa, tongue, floor of the mouth, and palate, for any newly developed lesions associated with the onset of COVID-19. The salivary flow was determined using the passive drool collection technique.

**RESULTS:**

Lip dryness, gingivitis, tongue lesions, and taste loss were the most commonly reported oral symptoms in patients with mild-to-moderate COVID-19. The most common general symptoms were tiredness and headache (63.9%), followed by dry cough, myalgia, sore throat, and fever. This study found 139 occurrences of oral symptoms, of which 52 were dry lips (27 tobacco non-users, and 25 tobacco users), and 11 were gingivitis (five non-users, and six tobacco users), and 12 tongue changes (eight non-users, and four tobacco users). Ageusia, or loss of taste sensation, was most commonly reported with or without other oral COVID-19 symptoms (55 occurrences: 36 non-users and 19 tobacco users). No significant differences were found in oral symptoms between tobacco non-users and tobacco users.

**CONCLUSIONS:**

There is a need to expand the routine examination protocol for patients during future respiratory pandemics, as monitoring oral health allows dentists to improve the management of oral sequelae during a pandemic.

## INTRODUCTION

Coronavirus disease (COVID-19) is a viral infection caused by the severe acute respiratory syndrome coronavirus 2 (SARS-CoV-2). COVID-19 imparts respiratory and extra-respiratory clinical signs and symptoms^1^. A surface protein of SARS-CoV-2 has a high binding affinity for angiotensin-converting enzyme-2 (ACE2) receptors. ACE2 is expressed on keratinized epithelial cell surfaces of the oral cavity, including the dorsum of the tongue, hard palate, and salivary glands. Viral invasion of oral tissues through this receptor causes several changes, including inflammatory reactions in the tongue mucosal membrane and salivary gland tissues^2^. The high expression levels could explain potentially observed oral symptoms in patients with COVID-19.

Several oral symptoms may be linked to the COVID-19, including tongue depapillation, candida-associated lesions, xerostomia, aphthous-like lesions, recurrent herpes virus infection, ulcers, blisters, gingivitis, gingival bleeding, necrotizing gingivitis, white/erythematous plaques, and salivary gland alterations. Loss of smell and taste with altered taste-bud sensitivity is also associated with COVID-19 oral lesions^3^.

The etiology of oral symptoms in patients with COVID-19 remains unclear; however, many possible molecular and cellular mechanisms have been suggested. Recent studies have indicated that: 1) the interaction between the virus and ACE2 receptors potentially interrupts cell integrity, inducing oral lesions; 2) the medications used to treat COVID-19 may trigger oral lesions, including herpes simplex virus, candida infection, ulceration, and gingivitis; and 3) oral lesions may develop due to the effect of SARS-CoV-2 on the immune system, increasing the risk of co-infection with opportunistic bacteria, fungi, and viruses. Oral lesions often resolve when COVID-19 symptoms subside^4^, confirming an association between COVID-19 and clinical oral symptoms.

Tobacco use is also a critical risk factor for the development of oral mucosal lesions ranging from benign mucosal changes to precancerous and cancerous lesions. Tobacco use is associated with periodontitis, halitosis, caries, and alveolar osteitis, resulting in a significant health burden. Smoking cigarettes often involves hand-to-mouth contact, and smokeless tobacco, including chewing tobacco, helps spread microorganisms when the user spits out saliva produced during the chewing process^5^. Moreover, tobacco use reduces the effectiveness of the immune system, making tobacco users more susceptible to diseases with poor prognoses^6^.

However, the prevalence of oral symptoms associated with COVID-19 onset among tobacco users remains unclear. This cross-sectional study investigated the oral symptoms in patients with mild-to-moderate COVID-19 to determine whether tobacco products intensify oral symptoms at disease onset.

## METHODS

### Study design and selection of patients

This cross-sectional study was conducted between January and March 2020. We selected a convenience sample from COVID-19 patients who were recruited to the Tetamman Primary Health Care Center based on their polymerase chain reaction (PCR)-positive results, which detected the presence of viral genetic material in nasopharyngeal or throat swabs. Patients were referred to the center to receive their medications. Our inclusion criteria were adult patients (aged ≥18 years), confirmed COVID-19 positive results, mild-to-moderate symptoms (fever, cough, sore throat, malaise, headache, muscle pain, nausea, vomiting, diarrhea, loss of taste and smell, and lack of shortness of breath, dyspnea, or abnormal chest imaging), no hospitalization, and enrollment within seven days of diagnosis. Patients were excluded if they were pregnant or lactating, had serious COVID-19 complications (e.g. severe pneumonia, severe dyspnea, increased respiratory rate >30 breaths/min, decreased oxygen saturation <93%), required hospitalization, or were on established antiviral, corticosteroid, antimicrobial, or immunosuppressive medications. When eligibility was determined, informed consent was obtained, the patient was interviewed, and an oral examination was conducted. Ethical approval was obtained from the Ethics Committee of the Directorate of Health Affairs, Ministry of Health, Jeddah, Saudi Arabia (protocol number: H-02-J-002; 1384). The study was conducted in accordance with the relevant guidelines and regulations of the Ministry of Health and the principles of the Declaration of Helsinki.

The power analysis in this study was computed using an alpha of 0.05 and a statistical power of 0.8 to estimate an effect size of 0.20 using the power program (G*Power software, version 3.1.9.7; Christian-Albrechts-Universität Kiel, Kiel, Germany).

### Data collection

Demographic data, medical history, and general COVID-19 symptoms were collected. We asked the patients about their perceptions of gustatory and olfactory dysfunction. Trained qualified dentists conducted meticulous examinations to assess the presence of oral lesions and conditions. The examination included inspection of the labial, buccal, and gingival mucosa, tongue, floor of the mouth, and palate for any newly developed inflammation, erythema, petechiae, ulcers, and macules. Tobacco-associated lesions (i.e. tobacco-induced melanosis) were excluded. The clinical diagnosis of oral mucosa was based on World Health Organization (WHO) criteria. The clinical diagnosis of mucosal lesions in the chewers was based on the criteria described by Zain et al.^7^ Data collection and clinical examination were performed by three examiners, and any abnormalities were reported after consultation and agreement among them.

Oral examinations were performed in a dental clinic under direct illumination built into the dental unit using a sterilized disposable mouth mirror and a probe. Strict precautions were taken, including wearing complete personal protective equipment, including disposable isolation gowns, sterile gloves, N95 masks, goggles, and face shields.

The salivary flow of all participants was determined using 50 mL calibrated Falcon sterile test tubes. Unstimulated whole-saliva samples were collected using the passive drool collection technique. Participants were asked not to talk, eat, drink, smoke, or chew anything for 30 min before giving the saliva samples. Participants were asked to swallow their saliva before the collection procedure began. Subsequently, they were asked to collect their saliva by drooling into a 50 mL sterile tube. The unstimulated salivary flow rate was considered normal when it was 0.25– 0.4 mL/min. Any reported flow <0.25 mL/min was considered low and a sign of oral dryness and salivary hypofunction.

### Statistical analysis

Data that were collected were edited and screened before entering entering into version 22 of the Statistical Package for the Social Sciences (SPSS Inc., Chicago, IL, USA). All statistical tests were two-tailed, and a p<0.05 was considered statistically significant. Descriptive statistics were used to compute the frequencies and percentages for all categorical variables, whereas means were used to describe the scale variables. Chi-squared and Fisher’s exact tests were used to assess the statistical significance of the associations between categorical variables. Backward logistic regression was used for multivariate analysis adjusting for age, sex, marital status, nationality, tobacco use, and chronic diseases were included in Step 1 of the analysis. Adjusted odds ratios (AOR) and their 95% confidence intervals (CIs) were calculated.

## RESULTS

This cross-sectional study included 122 participants (90 male and 32 female) diagnosed with COVID-19 with mild-to-moderate symptoms. The characteristics of the participants included in this study are presented in Table 1. Mean (SD) age was 35.4 (10.7) years for all the participants, 32.0 (10.0) years for females, and 36.6 (10.7) years for males. Most participants were aged 30–39 years, and the smallest group had participants aged >50 years. Most participants were male, married, and non-Saudi citizens. Diabetes was the most common chronic disease among study participants, followed by hypertension, high cholesterol levels, and asthma (Table 1). Approximately 38% of the participants were tobacco users, and the majority of them were male.

**Table 1:** Sample characteristics of non-hospitalized patients, aged ≥18 years, recruited to the Tetamman Primary Health Care Center with mild-to-moderate COVID-19 diagnosed by polymerized chain reaction ( PCR), between January and March 2020 (N=122)

*Characteristics*	*Overall (N=122) n (%)*	*Patients showing oral manifestations associated with COVID-19 (N=41) n (%)*
**Age** (years)		
18–29	39 (32)	11 (26.8)
30–39	44 (36.1)	14 (34.1)
40–49	25 (20.5)	11 (26.8)
≥50	14 (11.5)	5 (12.2)
**Sex**		
Male	90 (73.8)	30 (73.2)
Female	32 (26.2)	11 (26.8)
**Marital status**		
Single	40 (32.8)	12 (29.3)
Married	80 (65.6)	28 (68.3)
Divorced	2 (1.6)	1 (2.4)
**Nationality**		
Saudi	37 (30.3)	12 (29.3)
Non-Saudi	85 (69.7)	29 (70.7)
**Chronic disease**		
Diabetes	6 (4.9)	2 (4.9)
Hypertension	5 (4.1)	3 (7.3)
High cholesterol	2 (1.6)	1 (2.4)
Asthma	1 (0.8)	0 (0)

The overall results among tobacco non-users and tobacco users showed that tiredness and headache were the most common general symptoms reported in this study (63.9%), followed by dry cough, myalgia, sore throat, and fever. Approximately 50% of participants reported a loss of smell as a COVID-19 symptom. General symptoms were higher in non-users than in tobacco users, except for skin rashes and vomiting, which were higher in tobacco users (95.7% and 8.7%, respectively). However, we identified a nonsignificant difference in general symptoms between non-users and tobacco users (Table 2).

**Table 2:** General symptoms in non-hospitalized patients, aged ≥18 years, recruited to the Tetamman Primary Health Care Center with mild-to-moderate COVID-19 diagnosed by polymerized chain reaction (PCR), with and without tobacco habits, between January and March 2020 (N=122)

*General symptoms*	*Overall (N=122) n (%)*	*No habits (N=76) n (%)*	*Tobacco users (N=46) n (%)*	*Pearson χ²*	*p*
**Fever**				0.11	0.740
Yes	56 (45.9)	34 (44.7)	22 (47.8)		
No	66 (54.1)	42 (55.3)	24 (52.2)		
**Chills**				0.34	0.561
Yes	49 (40.2)	29 (38.2)	20 (43.5)		
No	73 (59.8)	47 (61.8)	26 (56.5)		
**Runny nose**				0.21	0.651
Yes	32 (26.2)	21 (27.6)	11 (23.9)		
No	90 (73.8)	55 (72.4)	35 (76.1)		
**Tiredness**				0.05	0.818
Yes	78 (63.9)	48 (63.2)	30 (65.2)		
No	44 (36.1)	28 (36.8)	16 (34.8)		
**Sore throat**				0.37	0.544
Yes	62 (50.8)	37 (48.7)	25 (54.3)		
No	60 (49.2)	39 (51.3)	21 (45.7)		
**Dry cough**				0.18	0.674
Yes	74 (60.7)	45 (59.2)	29 (63.0)		
No	48 (39.3)	31 (40.8)	17 (37.0)		
**Shortness of breath**				0.30	0.583
Yes	44 (36.1)	26 (34.2)	18 (39.1)		
No	78 (63.9)	50 (65.8)	28 (60.9)		
**Chest pain**				0.17	0.682
Yes	29 (23.8)	19 (25.0)	10 (21.7)		
No	93 (76.2)	57 (75.0)	36 (78.3)		
**Headache**				1.02	0.314
Yes	78 (63.9)	46 (60.5)	32 (69.6)		
No	44 (36.1)	30 (39.5)	14 (30.4)		
**Myalgia**				0.15	0.702
Yes	69 (56.6)	44 (57.9)	25 (54.3)		
No	53 (43.4)	32 (42.1)	21 (45.7)		
**Diarrhea**				0.67	0.413
Yes	27 (22.1)	15 (19.7)	12 (26.1)		
No	95 (77.9)	61 (80.3)	34 (73.9)		
**Skin rash**				1.10*	0.556
Yes	3 (2.5)	1 (1.3)	44 (95.7)		
No	119 (97.5)	75 (98.7)	2 (4.3)		
**Vomiting**				2.25*	0.197
Yes	6 (4.9)	2 (2.6)	4 (8.7)		
No	116 (95.1)	74 (97.4)	42 (91.3)		
**Nausea**				0.30*	0.765
Yes	13 (10.7)	9 (11.8)	4 (8.7)		
No	109 (89.3)	67 (88.2)	42 (91.3)		
**Red painful eye**				0.55*	0.474
Yes	8 (6.6)	4 (5.3)	4 (8.7)		
No	114 (93.4)	72 (94.7)	42 (91.3)		
**Loss of smell**				0.14	0.709
Yes	61 (50.0)	39 (51.3)	22 (47.8)		
No	61 (50.0)	37 (48.7)	24 (52.2)		

* Fisher’s exact test. Statistical significance at p<0.05.

The observed occurrences and sites of oral manifestations and lesions in mild-to-moderate COVID-19 patients are shown in Table 3. This study reported 139 occurrences of oral symptoms, of which 52 were dry lips (27 non-users and 25 tobacco users), and 11 were gingivitis (five non-users and six tobacco users). We also found 12 changes in the tongue (eight non-users and four tobacco users), including dry tongue, fissured tongue, geographic tongue, reddish macules in the tongue, mass on the tongue side, and tongue ulcers. Some patients experienced multiple symptoms. Ageusia, or loss of the sense of taste, was most commonly reported with or without other oral COVID-19 symptoms (55 occurrences: 36 non-users and 19 tobacco users), as shown in Table 3. Statistical analysis of reported oral symptoms showed no significant differences between non-users and tobacco users with regard to COVID-19 oral symptoms.

**Table 3:** Oral mucosal lesions in non-hospitalized patients, aged ≥18 years, recruited to the Tetamman Primary Health Care Center with mild-to-moderate COVID-19 diagnosed by polymerized chain reaction ( PCR), with and without tobacco habits, between January and March 2020 (N=122)

*Oral manifestations*	*Occurrence in tobacco users n (%)*	*Occurrence in tobacco non-users n (%)*	*p*
Dry lips	25 (54.3)	27 (35.5)	0.562
Dry buccal and labial mucosa	1 (2.2)	1 (1.3)	0.718
Small blisters in buccal and labial mucosa	0 (0.0)	1 (1.3)	0.435
Brown pigmentation (smoker melanosis)	1 (2.2)	0 (0.0)	0.197
Smokeless tobacco lesion	2 (4.3)	0 (0.0)	0.067
Gingival inflammation	6 (8.7)	5 (7.9)	0.876
White and erythematous gingiva	2 (4.3)	0 (0.0)	0.067
Dry tongue	3 (6.5)	3 (3.9)	0.524
Fissured tongue	0 (0.0)	1 (1.3)	0.435
Geographic tongue	0 (0.0)	1 (1.3)	0.435
Reddish macule in the tongue	0 (0.0)	1 (1.3)	0.435
Mass on the side of the tongue	0 (0.0)	1 (1.3)	0.435
Tongue ulcers	1 (2.2)	1 (1.3)	0.718
Dry mucosa on the floor of the mouth	0 (0.0)	1 (1.3)	0.435
Loss of taste	19 (41.3)	36 (47.4)	0.514

We found that 51% of non-users had normal salivary flow rates compared with 67% of tobacco users. However, 49% of non-users showed a low flow rate during saliva collection compared to 33% of tobacco users; however, the difference was not significant.

Backward logistic regression was used for multivariate analysis. Age, sex, marital status, nationality, tobacco use, and chronic diseases were included in the analysis. The analysis yielded a final significant model that included two variables. Women were significantly more likely to have oral manifestations compared to men (AOR=2.73; 95% CI: 1.08–6.90). There was no significant difference in oral manifestation between tobacco users and non-users (AOR=1.97; 95% CI: 0.84–4.64) (Table 4).

**Table 4:** Variables associated with oral manifestations in multiple logistic regression in non-hospitalized patients, aged ≥18 years, recruited to the Tetamman Primary Health Care Center with mild-to-moderate COVID-19 diagnosed by polymerized chain reaction (PCR), with and without tobacco habits, between January and March 2020 (N=122)

*Variable*	*B*	*Wald*	*p*	*AOR (95% CI)*
**Tobacco use status**				
User	0.682	2.4	0.117	1.97 (0.84–4.64)
Non-user ®				1
**Gender**				
Female	0.921	3.2	0.034	2.73 (1.08–6.90)
Male ®				1

AOR: adjusted odds ratio. Backward logistic regression was used for multivariate analysis. Age, sex, marital status, nationality, tobacco use, and chronic diseases were included in Step 1 of the analysis. ® Reference categories.

## DISCUSSION

The lack of sufficient information regarding the prevalence of oral symptoms in mild-to-moderate COVID-19 disease among tobacco users prompted a need to identify the oral symptoms in this patient group. Of the participants, 38% used different forms of tobacco, including conventional cigarettes, e-cigarettes, hookahs, and smokeless tobacco (e.g. chewing tobacco).

We included tobacco users in this study because previous studies have shown that tobacco use can make individuals more susceptible to COVID-19^8^. This may be because smoking habits facilitate cross-infection through contaminated fingers, cigarettes, pipes, and mouth, thereby increasing the risk of SARS-CoV-2 transmission. Hookah smoking involves the sharing of different mouthpieces, which facilitates SARS-CoV-2 transmission. Finally, COVID-19 can be transmitted via a viral load in excess saliva expectorated while chewing tobacco^8^.

In the present study, patients reported different general symptoms of COVID-19, including fatigue, headache, dry cough, myalgia, sore throat, fever, and loss of smell. This is consistent with several clinical studies that observed similar symptoms in mild and moderate cases of COVID-19 ^9^. In addition, 47% of tobacco users were male, and 13% were female. This is similar to the results of other studies in which predominant tobacco users were males^10^. However, Lima et al.^[Bibr cit0011]11^ showed a female predominance in tobacco use. The majority of the participants in the current study who used tobacco were aged 30–39 years. In contrast, other studies reported that the majority of the patients in the conventional smoking group were aged 50–65 years, whereas those aged 18–33 years dominated the smokeless tobacco group^12^.

The study findings suggest that the lips, gingiva, and tongue are the locations of oral manifestations of COVID-19. Moreover, single or multiple oral symptoms were observed in patients with COVID-19. These findings are consistent with previous reports that the tongue, gingiva, and lips are the main sites of oral manifestations of COVID-19^13-15^. The concurrent appearance of two or three oral symptoms can be attributed to ACE2 receptor expression in various oral tissues. High expression in the oral cavity is significantly associated with the oral manifestations of COVID-19, and the interaction between SARS-CoV-2 and this receptor is essential for inducing changes in the oral epithelium. Studies have reported that oral lesions are observed in 2–20% of patients with COVID-19^16,17^. Other studies have shown that oral lesions associated with the onset of COVID-19 resolve automatically when the infection subsides, indicating an association between the disease and reported oral symptoms^3^.

Oral symptoms reported in this study included dryness, inflammation, and ulcers. A recent study reported several ulcers during oral examinations in patients with COVID-19, which were related to the stress associated with the infection^18^. Ulcers are indirectly linked to COVID-19 by potentially triggering nervous habit consequences, increasing blood pressure, and poor diet^19,20^. The present results are also consistent with those of a recently published review, wherein oral symptoms, such as taste alterations and mucosal ulcerations, were identified during oral examination of patients with COVID-19^21^. Additionally, gingivitis was observed among the oral symptoms reported in this study. Regarding the periodontium, COVID-19 has been shown to affect the patient’s immune system, which is associated with: 1) an increase in the level of infectious agents that correlates with the development of periodontal conditions^22^, 2) high expression of cytokines, or ‘cytokine storm’, which may lead to tissue destruction^23,24^; and 3) increased fibrinogen degradation product levels as the coagulation cascade is activated during inflammation^25^.

Olfactory and gustatory dysfunctions were the most frequently reported symptoms in this study, independent of other oral manifestations of COVID-19. Song et al.^[Bibr cit0016]16^ reported a more frequent occurrence of gustatory dysfunction (21%) than olfactory dysfunction (11%) in patients with COVID-19, which is consistent with the results of the present study. Furthermore, the results here are consistent with previous studies reporting that gustatory dysfunction manifests in approximately 63%, 66%, 60.7%, and 45%^2,26,27^, of patients with COVID-19. The mechanisms underlying changes in smell and taste sensations caused by COVID-19 remain unclear. However, smell and taste dysfunctions may be attributed to the interaction of SARS-CoV-2 with the ACE2 receptor, which is broadly expressed by respiratory epithelial cells and oral mucosal tissues, predominantly in the tongue^28^.

Other hypotheses have suggested that the relationship between COVID-19 and loss of taste might be related to rhinitis, which initiates an inflammatory response that subsequently impairs normal taste bud function^29^. However, in many cases, loss of taste is not associated with nasal mucosal inflammation. The loss of taste in patients is linked to: 1) the inability to recognize food odor due to olfactory dysfunction, and 2) the failure to dissolve food molecules to enter the taste receptors due to saliva deficiency^30^. Conversely, olfactory dysfunction is caused by the mechanical obstruction of odorant transmission in the olfactory cleft due to mucosal inflammation, leading to olfactory epithelium degeneration and loss of smell^31^.

More than half of the participants retained a normal salivary flow rate during the SARS-CoV-2 infection period, regardless of tobacco use. However, approximately 49% of tobacco non-users and 33% of tobacco users exhibit low salivary flow rates. These results are consistent with those of previously reported cases of xerostomia^9^. Changes in salivary flow and composition in patients with COVID-19 may be explained by the damage to the epithelial cells of the salivary glands caused by SARS-CoV-2^32^. This result is similar to those of respiratory tract infections in which xerostomia has been reported^33^. As ACE2-positive cells are expressed in the salivary gland duct epithelial cells, it is hypothesized that viral infection activates pathological inflammatory lesions in the salivary glands, leading to lysis and epithelial cell damage and causing alterations in salivary flow^28^. Moreover, xerostomia experienced by COVID-19 patients may be caused by anxiety due to stressful conditions.

Clinical evidence has demonstrated that the oral cavity is the main site of SARS-CoV-2 entry. Therefore, the symptoms presented during COVID-19 onset are oral manifestations of COVID-19^34^, although this remains unclear. It has been hypothesized that the reported oral symptoms may be caused by COVID-19 disease, which promotes favorable conditions for the development of opportunistic viral or bacterial infections in the oral cavity. Therefore, it is crucial to maintain reasonable oral hygiene to control COVID-19 oral complications because of the established link between bacterial loads in the oral cavity and COVID-19 symptoms^35^.

### Limitations

All results reported in this article should be interpreted with caution. Taste loss could be subjective, as it was self-reported, and may not be diagnostic of this symptom. A more accurate classification of taste disorders may improve the measurement of this trait. The difficulty in recruiting patients with COVID-19 more than once due to their health conditions resulted in oral examinations being performed only once. Conducting oral examinations regularly every three days from the first day of onset to day 14 would provide a clearer picture of changes in oral health. Moreover, this study was conducted in one city in the Kingdom of Saudi Arabia, which may limit its generalizability. Another limitation is its cross-sectional design, which makes it impossible to prove causality between the variables. In addition, there is a lack of power in the comparison of tobacco users and non-users and the absence of comparisons with non-COVID-19 patients. Moreover, in such a small sample size, it would be preferable not to compare women and men unless a suitable sample size is established for both sexes.

## CONCLUSIONS

The COVID-19 pandemic caused by SARS-CoV-2 had a variety of consequences affecting oral health. Among the oral symptoms associated with mild-to-moderate COVID-19, dry lips, gingivitis, tongue lesions, and loss of taste were the most prevalent. However, there were no significant differences in oral symptoms between tobacco users and non-users with mild-to-moderate COVID-19. In dental clinics, meticulous oral examinations should be performed during any pandemic as part of the routine examinations to detect associated oral symptoms.

## Data Availability

The data supporting this research cannot be made available for privacy or other reasons.
