# Corrigendum: Oral symptoms potentially associated with mild-to-moderate COVID-19 in tobacco users

**DOI:** 10.18332/tid/200457

**Published:** 2025-03-12

**Authors:** Hanaa E. Alkharobi, Manar M. Alzahrani, Shatha Bamashmous, Abdullah Alghamdi

**Affiliations:** 1 Department of Oral Biology, College of Dentistry, King Abdul-Aziz University, Jeddah, Saudi Arabia; 2 Department of Oral and Maxillofacial Prosthodontics, Faculty of Dentistry, King Abdulaziz University, Jeddah, Saudi Arabia; 3 Department of Periodontology, Faculty of Dentistry, King Abdulaziz University, Jeddah, Saudi Arabia; 4 Ministry of Health, Jeddah, Saudi Arabia

**Keywords:** COVID-19, oral, manifestations, tobacco products, ageusia

Corrigendum on:

Oral symptoms potentially associated with mild-to-moderate COVID-19 in tobacco users

By Hanaa E. Alkharobi, Manar M. Alzahrani, Shatha Bamashmous, Abdullah Alghamdi

Tobacco Induced Diseases, Volume 22, Issue May, Pages 1–10

Publish date: 13 May 2024

DOI: https://doi.org/10.18332/tid/186531

In the updated version of the article, the authors Manar M. Alzahrani, Shatha Bamashmous, Abdullah Alghamdi were added after the author Hanaa E. Alkharobi as the 2nd, 3rd and 4th author and their affiliations superscripts were inserted accordingly. All the authors have completed and submitted the ICMJE Form for Disclosure of Potential Conflicts of Interest and none was reported. All the authors agree that the added authors meet all four criteria for authorship and that the omission of their names during publication was inadvertent.

Finally, the copyright has changed to “^©^ 2024 Alkharobi H.E. et al.”, the “Acknowledgements” section to “The authors thank the Ministry of Health for their cooperation and assistance in conducting the study.”, the Conflicts of Interest section to “The authors have completed and submitted the ICMJE Form for Disclosure of Potential Conflicts of Interest and none was reported.”

The mentioned changes have also been completed online.

